# Optimization of Mass and Light Transport in Nanoparticle-Based Titania Aerogels

**DOI:** 10.1021/acs.chemmater.3c01218

**Published:** 2023-09-20

**Authors:** Fabian Matter, Markus Niederberger

**Affiliations:** Laboratory for Multifunctional Materials, Department of Materials, ETH Zurich, Vladimir-Prelog-Weg 5, 8093 Zurich, Switzerland

## Abstract

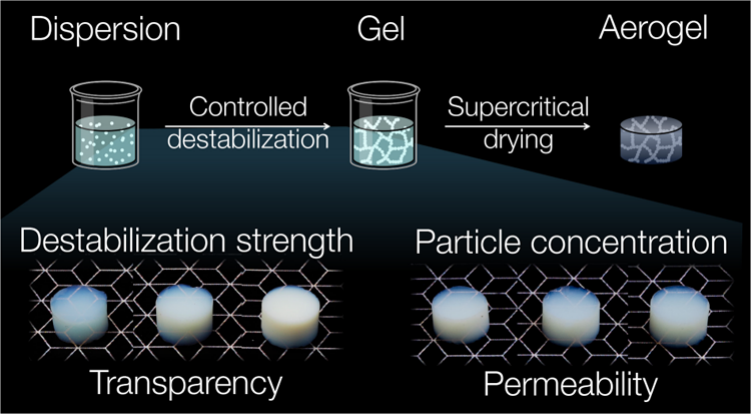

Aerogels composed of preformed titania nanocrystals exhibit a large surface area, open porosity, and high crystallinity, making these materials appealing for applications in gas-phase photocatalysis. Recent studies on nanoparticle-based titania aerogels have mainly focused on optimizing their composition to improve photocatalytic performance. Little attention has been paid to modification at the microstructural level to control fundamental properties such as gas permeability and light transmittance, although these features are of fundamental importance, especially for photocatalysts of macroscopic size. In this study, we systematically control the porosity and transparency of titania gels and aerogels by adjusting the particle loading and nonsolvent fraction during the gelation step. Mass transport and light transport were assessed by gas permeability and light attenuation measurements, and the results were related to the microstructure determined by gas sorption analysis and scanning electron microscopy. Mass transport through the aerogel network was found to proceed primarily via Knudsen diffusion leading to relatively low permeabilities in the range of 10^–5^–10^–6^ m^2^/s, despite very high porosities of 96–99%. While permeability was found to depend mainly on particle loading, the optical properties are predominantly affected by the amount of nonsolvent during gelation, allowing independent tuning of mass and light transport.

## Introduction

Aerogels consist of a finely branched three-dimensional network that offers very high porosity and a large internal surface area, making them particularly attractive for applications such as detection and sensing,^1,2^ filtration, and catalysis.^3−5^ Aerogels have been manufactured for decades using conventional sol–gel chemistry,^6,7^ a process that is, however, limited when it comes to design flexibility. Recently, an alternative method has emerged in which aerogels are assembled from presynthesized nanoparticles.^8,9^ Using this approach, a variety of different materials has already been processed into aerogels, including metals,^10−13^ their oxides,^14−21^ nitrides,^22^ phosphides,^23,24^ chalcogenides,^25−27^ fluorides,^28^ and combinations thereof. As the properties of the nanoscale building blocks are typically preserved during the assembly process, nanoparticle-based aerogels can be precisely tailored to a specific application, not only in terms of composition but also with respect to the size, shape, surface chemistry, and crystallinity of the underlying building blocks.^8^

By assembling titania nanocrystals, for example, translucent aerogels can be produced with a surface area of up to 500 m^2^/g and porosity of up to 99%.^29,30^ The high degree of crystallinity combined with the translucency and open porosity make these structures ideally suited for gas-phase photocatalysis. The photoactive titania network can be further decorated with selected cocatalysts to tailor the photocatalyst composition to specific reactions. Several studies have demonstrated the potential applications of these titania aerogels, including photocatalytic conversion of carbon dioxide or hydrogen production from organic feedstocks.^31−34^

Recent studies have focused on exploiting new building blocks and enhancing photocatalytic performance by tuning their composition. In contrast, little attention has been paid to modifications at the structural level, although microstructure affects crucial elements for photocatalysis, such as light transmission and mass transfer rate. While efficient mass transport is typically easy to accomplish for powders and films due to the short transport paths, the pathways in three-dimensional aerogel catalysts are several orders of magnitude longer. To identify and tackle potential transport limitations in such porous three-dimensional architectures, knowledge about gas permeability and the prevailing transport mechanism is required.^35^ A few studies already exist on mass transport through conventional sol–gel-derived silica^36−41^ and carbon aerogels.^39,42−44^ However, no data exist on this subject for nanoparticle-based aerogels.

This study thus investigates the gas transport through nanoparticle-based titania aerogels in detail. We further show how structural modifications can be realized to control fundamental properties, such as gas permeability and light transmittance. To this end, a series of centimeter-sized titania aerogels with different porosities and optical properties are prepared through an optimized fabrication process and studied by steady-state permeability measurements and light scattering methods. Complementary characterization by nitrogen gas sorption and scanning electron microscopy also provides valuable insights into the formation mechanism of this fascinating class of materials.

### Theoretical Background

Mass transport in porous materials is typically divided into two categories: advective and diffusive mass transport. Advection is based on the collective motion of molecules caused by an external force. It dominates in pressure-driven mass transport through pores with sizes of hundreds of nanometers and larger. Diffusion, on the other hand, is the result of the thermal motion of individual molecules, which leads to a net flux along a concentration gradient. This type of mechanism typically prevails in materials with smaller pore sizes where pressure-driven advection is strongly impaired. Aerogels can have a broad pore size distribution, extending from the subnanometer range to several hundred nanometers. Mass transport can thus take place either by advection, by diffusion, or through a combination thereof. The extent to which the two transport types contribute to overall gas transport can be elucidated by permeability measurements. The gas permeability of a material can be determined experimentally by applying a pressure gradient along the sample axis and measuring the resulting gas flow
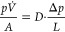
1with *p* being the pressure and  the volumetric flow rate at the measuring point, *A* the cross-sectional area of the sample, *D* the permeability, Δ*p* the pressure difference, and *L* the sample length.

Permeability is determined by the pore structure, fluid properties, and, depending on the type of transport, different experimental parameters. To relate the permeability to structural properties and experimental conditions, aerogels are typically modeled as an array of parallel cylindrical tubes.^45^ Advective flow through such a cylindrical pore is given by the Hagen–Poiseuille equation for compressible gases^46^
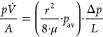
2with *A* being the cross-sectional area of the pore, *r* the pore radius, μ the dynamic viscosity, and *p*_av_ the average between the inlet and outlet pressure. Pressure-driven advective mass transport scales with the square of the pore radius and becomes very ineffective for materials with small pore sizes. Due to the compressibility of gases, the pressure gradient also results in a concentration gradient and thus a net diffusive flux across the sample. The transport rate for ordinary diffusion depends on the thermal velocity and the mean free path of the gas molecules.^46^ The mean free path is the average distance a molecule travels between collisions and depends on the number density of molecules and, thus, on pressure. In porous materials with pore sizes comparable to or smaller than the mean free path, molecule-wall collisions dominate over molecule–molecule interactions, making the diffusion rate no longer pressure-dependent. This type of transport is known as Knudsen diffusion. For an ideal gas, Knudsen diffusion through a cylindrical pore is given by^46^
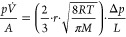
3where *A* is the cross-sectional area of the pore, *r* is the pore radius, *R* is the universal gas constant, *T* is the temperature, and *M* is the molecular mass of the gas molecule.

For pure diffusive flow, the permeability scales with the inverse of the square root of molar mass, while for advective flow, the permeability is proportional to the average pressure and the inverse of the dynamic viscosity of the gas. By measuring the permeability of a sample with different gases and at different pressures, the mass transport mechanism can be elucidated. Once the primary transport mechanism is identified, the measured permeability can be related to the pore size.

## Experimental Section

### Chemicals & Materials

Titanium(IV) tetrachloride (99.9% trace metal basis), benzyl alcohol (puriss., 99–100.5% (GC)), tris(hydroxymethyl)-aminomethane (Trizma base, puriss., ≥99.7%), ethanol (absolute ≥99.8% for analysis), diethyl ether (puriss., ≥99.8%), *n*-hexane (≥97.0% for HPLC), acetone (≥99.8% for HPLC), and Drierite (without indicator, 4 mesh) were purchased from Sigma-Aldrich/Merck. RTV Silicon Elastosil E43 was purchased from Wacker Chemie AG. Nitrogen 4.5, helium 4.6, oxygen 5.0, and carbon dioxide 3.0 were purchased from PanGas AG Switzerland, and sulfur hexafluoride 3.0 was purchased from Linde Gas. All chemicals were used as received without further purification.

### Sample Preparation

#### Synthesis of TiO_2_ Building Blocks

Anatase titania nanocrystals with a size of 3–3.5 nm were prepared by a modified nonaqueous synthesis route based on previously published protocols.^47−49^ In the first step, benzyl alcohol (160 mL) was filled into a 250 mL round-bottom flask and heated to 120 °C in a preheated oil bath. Meanwhile, titanium tetrachloride (8 mL, 73 mmol) was added dropwise to ice-cooled ethanol (24 mL, 411 mmol) in another 250 mL round-bottom flask under constant stirring at 200 rpm over 1–2 min. The addition rate was adjusted so that the released hydrochloric acid fume continuously redissolved in cold ethanol instead of escaping the flask. After addition, the greenish-yellow viscous solution was stirred for another 5 min. In a second step, mortared Trizma (728 mg, 6 mmol) was dissolved in the hot benzyl alcohol over 1 min before the cold ethanolic precursor solution was added. The reaction vessel was closed with a perforated lid to avoid overpressure, and the solution was kept for another 2 h at 120 °C under constant stirring at 500 rpm. During this period, the reaction solution gradually changed from clear yellow to translucent and eventually to milky, indicating the formation of titania nanoparticles. The reaction mixture was finally cooled to room temperature in a water bath.

For washing, 24 mL aliquots of the reaction solution were mixed with diethyl ether (20 mL) to precipitate the particles. The white precipitate was collected by centrifugation for 5 min at 4000 rpm and washed three times with diethyl ether (30 mL) and subsequently three times with hexane (30 mL). For each washing step, the wet precipitate was mixed with fresh solvent, shaken vigorously, and centrifuged for 1 min at 4000 rpm, before the clear supernatant was discarded. Finally, the precipitate was suspended in hexane (30 mL) before water (4 mL) was added to extract the nanoparticles. Upon gentle shaking, all nanoparticles were transferred to the aqueous phase, leaving behind a clear hexane supernatant. After centrifugation for another 2 min at 4000 rpm to improve phase separation, the clear aqueous dispersion was separated from the hexane phase with a syringe and passed through a syringe filter (PTFE, 0.45 μm) to remove any dust particles. The aqueous dispersions of one synthesis batch were combined and stored in a closed vessel at room temperature for 24 h before being used as stock solution for gel preparation. For longer-term storage, the colloidally stable dispersion was kept at 4 °C.

#### Preparation of Titania Gels and Aerogels

The gelation of the aqueous titania dispersion was induced by nonsolvent addition and subsequent heating, similar to previously published protocols.^29,30^ Two series of samples with varying solid fractions (12–100 mg/mL) and different quantities of nonsolvent (0–72 vol %) (see Table S1, Supporting Information) were produced from the same stock dispersion as follows: First, different ratios of acetone/water were premixed in a vial and cooled to their freezing point in liquid nitrogen. Second, the desired amount of aqueous titania stock solution was added under vortex before the clear mixture was again cooled close to its freezing point and drawn up with a 10 mL syringe. Complete freezing during any of the preparation steps was avoided, although multiple freeze–thaw cycles did not affect the stability of the dispersion or the gelation process. Third, the dispersion was degassed by closing the syringe inlet and pulling the plunger to twice the liquid volume. After shaking, the evolved gas was released and the degassing procedure was repeated two more times. To produce 8 similar samples, 8 × 0.4 mL of this degassed and still cold dispersion was molded into an 8 × 2 mL syringe with a cutoff tip, sealed with Parafilm, and gelled in a ventilated oven using the conditions indicated in Table S1 (SI).

The resulting gels were finally demolded in solvent baths (1.5 mL per sample in 5 mL vials) with the same acetone/water ratios as used for gelation. The vials with the immersed gels were stored overnight at 60 °C in a sealed vessel for aging. The bottom of this outer vessel was filled with the corresponding water/acetone mixture to prevent drying of the gels.

After this aging step, the aqueous pore fluid was gradually replaced with acetone via a vapor diffusion technique. To this end, the liquid in the outer vessel was replaced with pure acetone before the vessel was sealed and stored at 60 °C for another 24 h. During this time, vapor diffusion of acetone from the outer vessel into the vials resulted in a gradual increase in acetone concentration from the initial 18–63 vol % to approximately 65–90 vol %. The liquid in the vials was reduced to 1.5 mL by decantation, and the procedure was repeated with fresh acetone once more. As a final step, the solvent bath in the vials was replaced with pure acetone and left for 8 h before the samples were stored overnight in an acetone bath over Drierite.

After solvent exchange, the samples were transferred to small baskets and loaded into a supercritical drying chamber (E3100, Quorum Technologies) cooled to 5 °C. The chamber was filled with liquid CO_2_ and then five times emptied to half-capacity and refilled. This replacement procedure was repeated two more times, with a 30 min soak time in between, to exchange all of the pore fluid for liquid CO_2_. After the third cycle, the chamber was heated to 42 °C to reach the supercritical state of CO_2_. Once the temperature and pressure stabilized, the chamber fluid was kept in the supercritical state for 1 h at 100 bar. The pressure was finally reduced to atmospheric pressure over approximately 20 min before the aerogels were removed and stored in ambient atmosphere overnight. The supercritical drying procedure was performed separately for each of the two series.

### Characterization

#### Density Determination

The density of the aerogel monoliths was determined by dividing the weight of the sample by its volume. The volume of the cylindrical samples was calculated from their diameter and height. The curvature of the meniscus formed during molding was determined photographically and included in the calculation as a spherical segment (see the SI for details).

#### Gas Sorption Measurements

Nitrogen gas sorption experiments were performed on a Quantachrome autosorb iQ at 77 K. Specific surface area, pore size distribution, and pore volume were determined by density functional theory (DFT) using a nonlocal density functional theory (NLDFT) equilibrium model for silica with cylindrical pores. Prior to analysis, about 20 mg of sample was degassed at 100 °C for at least 20 h. The total pore volume *V*_tot_ and macropore volume *V*_macro_ were calculated according to
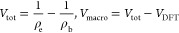
4+5with ρ_e_ being the effective density of the aerogel (without adsorbed water), ρ_b_ the bulk density of anatase titania = 3.89 g/cm^3^, and *V*_DFT_ the pore volume obtained by DFT analysis of gas sorption isotherms. The effective aerogel density was calculated by multiplying the apparent aerogel density by a factor of 0.89 that was determined by averaging the relative mass losses of all samples during outgassing. The average pore size *D*_av_ was determined by calculating the hydraulic diameter for channels with nonuniform and noncircular cross-sectional areas^45^
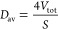
6with *V*_tot_ being the total pore volume described above and *S* the specific surface area determined by DFT analysis of gas sorption data (see Table S2 in the SI for numerical values).

#### Light Attenuation Measurements

The optical properties of gels with different solid fractions (12–100 mg/mL) and acetone concentrations (0–63 vol %) were studied using a Jasco V-670 spectrometer. Gel samples were prepared as described above (see Table S1, Supporting Information), except that the cold and degassed dispersion was filled into a PS semi-microcuvette (1.5 mL, BRAND), sealed with Parafilm, and gelled in the cuvette. The light attenuation of the gels was measured in transmission at 25 °C with an optical path length of 10 mm. Pure water–acetone mixtures were used as blanks.

#### Scanning Electron Microscopy

Scanning electron microscopy (SEM) images were recorded on a Zeiss Gemini 2 instrument operated at 5 kV using an in-lens detector. Small aerogel pieces were placed on conductive carbon tape and sputtered with 2 nm of Pt by a Safematic sputter coater CCU-010.

#### Permeability Measurements

For permeability measurements, the aerogel samples were sealed on a perforated PMMA disc by applying RTV silicone (Elastosil E43, Wacker) onto the side of the cylinders (see Figure S2 in the SI). The samples were stored for at least 2 h before measurement to allow the silicone to cure. The sealed samples were analyzed in a custom-made permeability setup (see the Results section) consisting of a gas supply, differential pressure controller (Alicat Scientific), sample cell, and flow meter (Alicat Scientific). Prior to measurement, the system was degassed and purged with the analysis gas. Permeability data were collected by applying different pressure gradients along the sample axis and recording the resulting steady-state volumetric flows. The volumetric flow rate was recorded at the outlet each second and converted to a volumetric flow rate at standard temperature and pressure (STP: 298.15 K, 101 325 Pa), which we refer to as the mass flow rate. The outlet was connected either to the atmosphere or to a 25 L vacuum vessel to provide quasi-steady outlet pressures for permeability studies below 1 atm. In a typical run, the pressure was increased from 0 to 300 mbar in 25 mbar steps, with an equilibration time of 60 s for each step. For experiments below ambient pressure, the equilibration time was set to 180 s. The steady-state mass flow rate was determined by averaging the last 10 data points of each step. Multiple permeability curves were recorded for each sample to evaluate the stability and exclude anomalies in the data. Each sample was measured in triplicate, omitting samples with drifting data or abrupt cracking.

Permeability was calculated from the permeability data using linear regression analysis
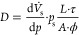
7where *D* is the permeability,  is the volumetric flow rate at standard temperature and pressure (*T*_s_ = 298.15 K, *p*_s_ = 101 325 Pa) measured at the outlet, Δ*p* is the applied differential pressure, *L* is the sample length in the flow direction (see the SI for more details), τ is the tortuosity factor set to unity, *A* is the cross-sectional area of the sample, and ϕ is the porosity of the sample, which was calculated by
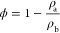
8with ρ_a_ being the apparent density of the aerogel and ρ_b_ the bulk density of anatase titania = 3.89 g/cm^3^ (see Table S2 in the SI for numerical values).

## Results & Discussion

### Fabrication of Titania Gels and Aerogels

**Figure 1:**
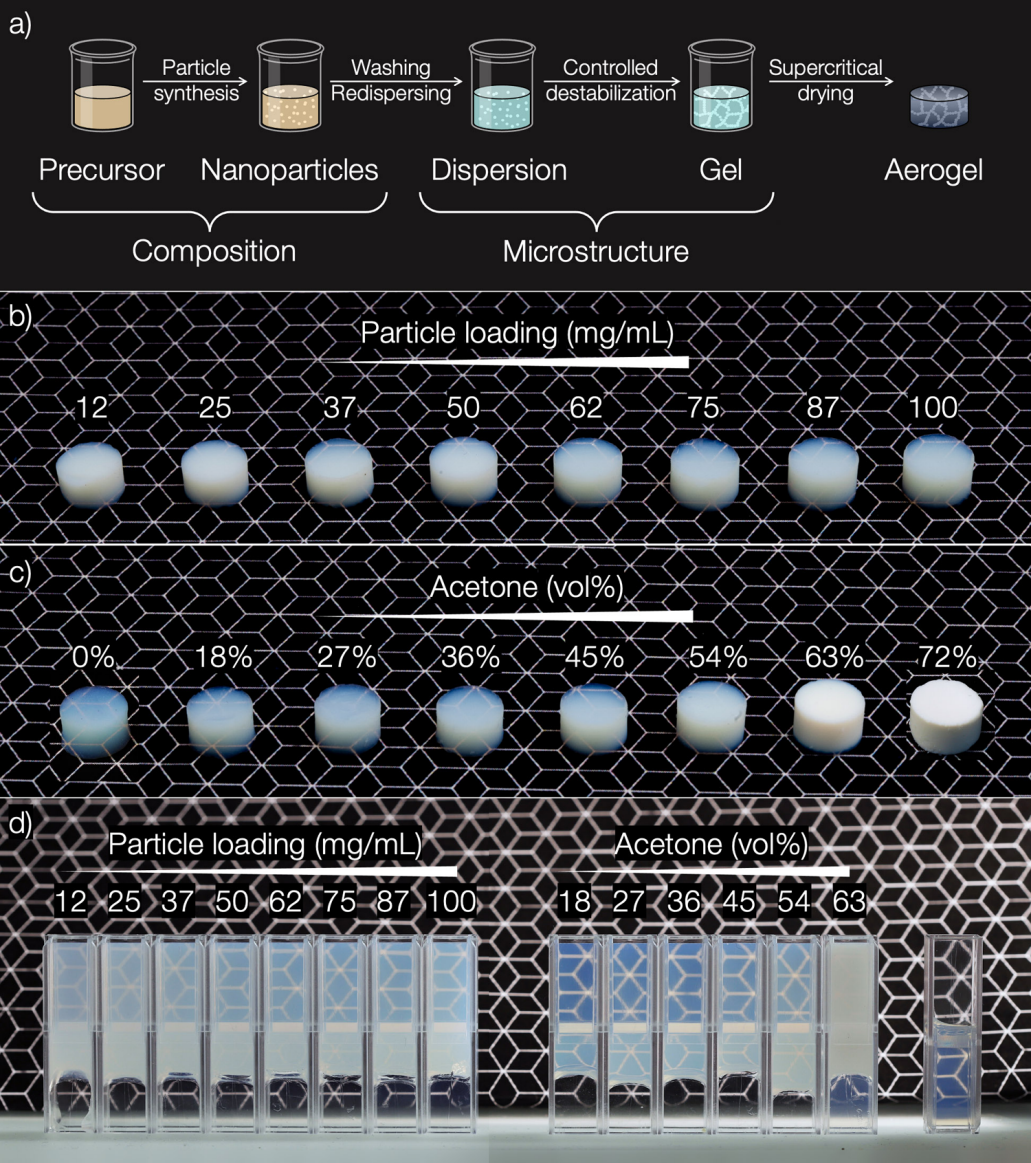
(a) Building block approach for the preparation of nanoparticle-based titania aerogels. First, titania nanoparticles are synthesized, washed, and redispersed to form a colloidally stable dispersion. Second, the dispersion is destabilized by nonsolvent and temperature to induce self-assembly into a gel. The pore liquid of the gel is finally exchanged by acetone and removed by supercritical drying, leaving behind a nanoparticle-based aerogel. The composition, size, and shape of the building blocks can be controlled in the synthesis step, whereas the microstructure of the network can be controlled during the assembly process. (b) Nanoparticle-based titania aerogel monoliths prepared from dispersions with different particle loadings (12–100 mg/mL) destabilized with a constant amount of nonsolvent (54 vol % acetone). (c) Nanoparticle-based titania aerogel monoliths prepared from a dispersion with a constant loading (75 mg/mL) destabilized by varying amounts of nonsolvent (0–72 vol % acetone). (d) Corresponding titania gels molded into UV–vis cuvettes for optical analysis together with the initial dispersion as a reference (far right).

For this study, titania gels and aerogels with different porosities and transparency were prepared using a building block approach (Figure 1). The starting point was a colloidally stable dispersion of charge-stabilized titania nanoparticles, which was gelled by the addition of nonsolvent and increase in temperature, analogous to previously published protocols,^29,30^ yet with some significant improvements to extend the range of opacities and loadings while maintaining high monolith quality.

Previous studies achieved gelation of titania dispersions primarily by the addition of ethanol and subsequent heating to 60 °C.^30−32,34^ Experiments with different amounts of ethanol revealed that the gel transparency decreased with increasing ethanol concentration. Nevertheless, it was not possible to cover the entire range of light transmittance with ethanol due to its limited destabilizing power, as high amounts of ethanol led to a strong dilution of the dispersion and prevented the formation of a space-spanning network. Follow-up studies revealed that most water-miscible solvents can induce gelation, including ethanol, acetone, isopropanol, tetrahydrofuran, dimethylformamide, *N*-methylpyrrolidone, acetonitrile, tert-butanol, 1,4-butanediol, triethylene glycol, and tetraethylene glycol. In general, solvents with lower dielectric constants exhibited higher destabilization power, similar to studies on charge-stabilized metal oxide and fluoride nanoparticles.^28^ This observation is expected, as the electrostatic repulsion between the nanoparticles is more short-ranged in media with low dielectric constant.^8^ Nonetheless, aqueous mixtures with different solvents but the same dielectric constant did not always result in the same degree of destabilization, suggesting that destabilization is more complex and relies not only on the decrease in dielectric constant but also on other factors such as polarity. Among the solvents tested, acetone was selected as the nonsolvent for further investigations because of its medium destabilizing power, giving access to aerogels covering the entire range of optical properties within a reasonable concentration range. To identify ideal gelation conditions, a series of experiments with different acetone fractions and particle loadings were performed.

Experiments with different acetone concentrations revealed that the optical appearance of the gels could be controlled from highly transparent to almost completely opaque within a range of 0–70 vol % acetone. Above a threshold value of 70–75 vol % acetone, the nanoparticles started to precipitate rather than gel, irrespective of the particle concentration. In addition, the acetone concentration was also found to have a strong impact on the gelation rate. Low acetone fractions (0–40 vol %) resulted in a relatively slow gelation process that could only be completed at elevated temperatures. In comparison, strongly destabilized (≥60 vol %) dispersions gelled within seconds even at room temperature, preventing homogeneous mixing and proper molding, which resulted in gels and aerogels with poor mechanical properties. To circumvent the problem of premature gelation at high acetone concentrations, acetone was added to the dispersion at sub-ambient temperatures, which completely suppressed gelation and allowed homogeneous mixing even at very high acetone concentrations well above 75 vol %. After molding, gelation was induced by heating the dispersion to ambient temperature. In this way, high-quality aerogel monoliths with high opacity can be obtained which are not accessible via previously reported room-temperature routes. We believe that the suppression of gelation at low temperatures arises from the increase in the dielectric constant of the cold mixture,^50^ which stabilizes the dispersion and counteracts the destabilization by the nonsolvent.

Investigations with differently diluted dispersions showed that particle loading primarily affects the gelation rate but only has a minor effect on the optical appearance. Moderately destabilized dispersions with high particle fractions gelled within minutes, whereas dispersions with low particle concentrations required hours or elevated temperatures to form a stable gel. Noteworthy, transparent gels with loadings down to 3 mg/mL could be produced in the course of this work, which corresponds to an exceptionally high theoretical porosity of 99.9%. Unfortunately, we were not able to produce aerogel monoliths with the given dimensions from gels with loadings lower than 10 mg/mL due to the low mechanical stability of the network. Moreover, samples with low loadings (10–40 mg/mL) experienced severe shrinkage during solvent exchange, whereas those with low acetone (0–30 vol %) fractions tended to swell or even dissolve during this step. To overcome these limitations, an additional aging step was implemented, and solvent exchange was performed at elevated temperature which reduced the linear shrinkage to 9–18% (see Table S2 in the SI).

The optimized manufacturing route described above enables the production of centimeter-sized titania aerogels with varying opacities and porosities in high quality and high yield. To study the effect of particle concentration and destabilization strength on the macroscopic properties, in particular on the optical transmission and gas permeability, two series of gel and aerogel samples were prepared with different particle loadings and nonsolvent fractions. In the first series, the particle concentration was varied between 12 and 100 mg/mL keeping the acetone fraction constant at 54 vol % (Figure 1b,d). In the second series, the particle loading was kept constant at 75 mg/mL, while the acetone fraction was varied between 18 and 63 vol % (Figure 1c,d). Samples prepared with 0 and 72 vol % acetone were not considered in further investigations due to swelling during solvent exchange and poor mechanical properties, respectively.

### Optical Characterization

**Figure 2:**
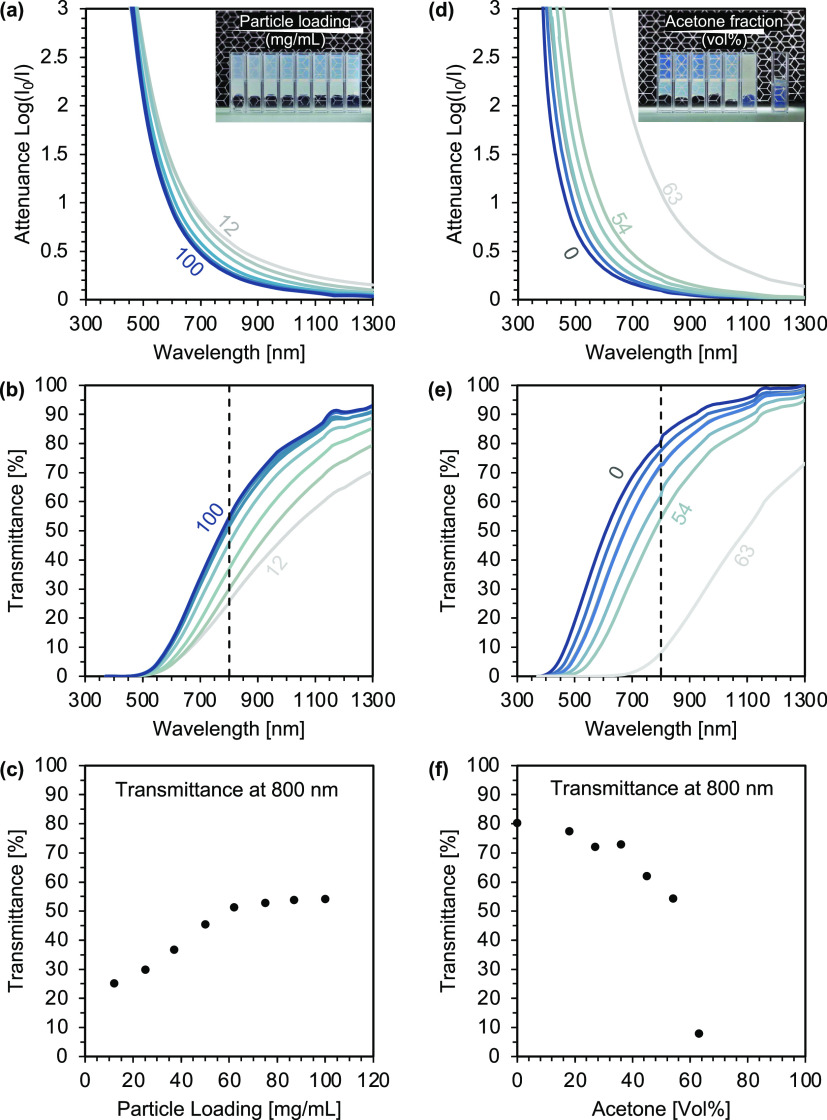
Effect of particle loading and acetone concentration during destabilization on the optical characteristics of titania gels. Left: Light attenuance and transmittance of gels prepared with varying particle loadings (12–100 mg/mL) and constant acetone fraction (54 vol %). Right: Light attenuance and transmittance of titania gels with constant loading (75 mg/mL) and varying acetone fractions (0–63 vol %) with 0 vol % being the initial dispersion. Optical path length: 10 mm.

Efficient light utilization is a key element for high photocatalytic performance, as light induces both charge carrier generation and local heating effects on the side of the cocatalyst. Understanding light propagation within a photocatalyst enables the optimization of reactor designs and catalyst geometries to maximize the interaction with light. The optical characteristics of titania gels (Figure 1d) were assessed by measuring their light attenuation using a UV–vis spectrometer. Gels have been used for this study as aerogels are fragile and difficult to handle, especially at low particle loadings. Figure 2 displays the optical attenuance and transmittance of the gels prepared with different particle loadings and destabilization strengths. The observed light attenuation primarily results from scattering effects as titania shows negligible absorption and luminescence in the visible light range. All samples show a decay in attenuance with increasing wavelength, with blue light being more strongly scattered than red light. This phenomenon is characteristic of samples with features much smaller than the wavelength of light and can be attributed to Rayleigh scattering.

By variation of the amount of acetone during destabilization, the optical properties of titania gels can be controlled over a wide range (Figure 2, right). Low acetone concentrations result in gels with an optical transmission similar to the initial dispersion (0 vol %), whereas high acetone fractions lead to strongly scattering samples with almost zero light transmission (Figure 2f) within the visible light spectrum. This observation agrees well with studies on CdS gels,^51^ in which CdS nanoparticles were assembled into volume-spanning networks with different degrees of opacity by varying the amount of destabilizer and can be explained by the variation in aggregation kinetics. The formation of titania gels from primary nanoparticles likely proceeds via the formation of secondary clusters, which, in turn, interconnect into a space-spanning network over time. In the aggregation kinetics of nanoparticles into clusters, two limiting regimes are generally distinguished depending on the strength of destabilization: Diffusion-limited cluster aggregation (DLCA) and reaction-limited cluster aggregation (RLCA).^52,53^ DLCA dominates when the particles are poorly stabilized and coalesce after the first collision with each other. As a result, the particles attach more frequently in the outer sphere of the clusters, resulting in more open and tenuous assemblies. In the case of RLCA, the particles are rather well stabilized and require multiple collisions before they stick to each other, which increases the probability that the particles also attach to the inner sphere of the cluster, resulting in more compact structures. Accordingly, high acetone concentrations cause the formation of larger and more open secondary clusters by DLCA, resulting in stronger scattering and thus a higher opacity. However, when the acetone threshold is exceeded, the attractive force between the branches of a cluster becomes too strong, causing the finely branched clusters to collapse and precipitate. Both the microscopy images and the gas sorption measurements point to such a formation mechanism, as we discuss later.

Following the theory of particle aggregation, the gels with different particle loadings are expected to have similar optical properties because they were destabilized under the same conditions, which should result in similar cluster structures.^54,55^Figure 2c shows that this is indeed the case for particle loadings above 50 mg/mL. However, at lower loadings, the optical transmittance starts to decrease. In addition, the attenuation decay is less pronounced for these samples, which manifests itself in a slightly grayish rather than bluish appearance. The increased opacity can be explained by the appearance of larger features such as pores that form between loosely packed clusters at low cluster concentrations. Macroscopically, voids act like additional scattering centers reducing the transmitted light.^21,56^ The grayish appearance, on the other hand, can be attributed to contributions from Mie scattering, which occurs when the feature size approaches the wavelength of visible light. The presence of such large pores was confirmed by microscopy images, as we discuss later. Unlike Rayleigh scattering, which is strongly dependent on wavelength and decreases with the fourth power of the wavelength, Mie scattering acts more uniformly over all wavelengths of visible light. Fitting the light attenuation data for both series revealed that gels with high particle loadings (≥50 mg/mL) follow a power law decay with an exponential factor between 4.2 and 4.4 (see Figure S4 in the SI), which is close to the expected theoretical value for pure Rayleigh scattering. For lower loadings (<50 mg/mL), however, the scattering decays with an exponential factor of only 2.9–3.7, indicating the presence of Mie scattering. Such a transition from Rayleigh scattering to Mie scattering was also observed in recent studies on sol–gel-derived silica aerogels.^21,56^ Although the transition occurred at slightly lower gel densities, its origin was also attributed to the presence of larger pores.

The analysis presented above has shown that the optical properties of nanoparticle-based titania gels can be tuned over a wide range by adjusting the nonsolvent fraction in the destabilization step. Particle loading, on the other hand, had little effect on the optical properties but is an effective tool to tune the porosity and thus the pore size, which dictates the rate of mass transfer. The effects of these two parameters on mass transport through nanoparticle-based titania aerogels are examined in detail in the following section.

### Gas Permeability Measurements

**Figure 3:**
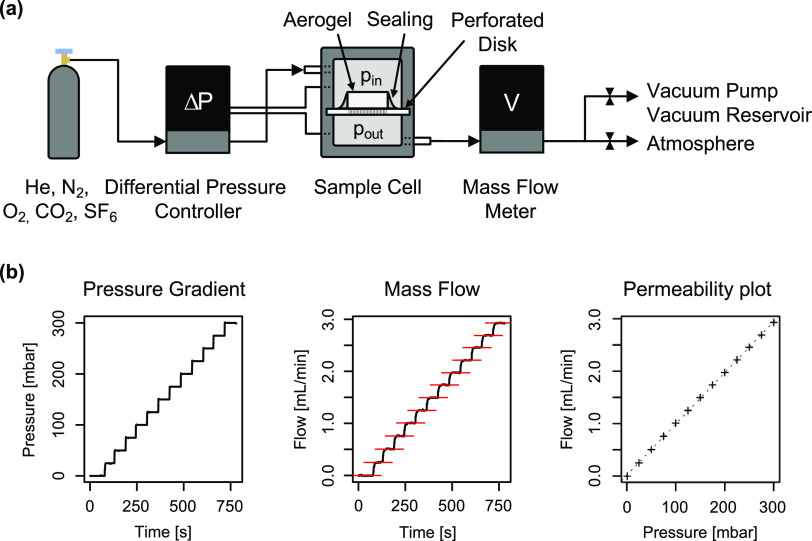
(a) Gas permeability measurement setup consisting of a gas supply, differential pressure controller, sample cell, and mass flow meter. The outlet is either connected to the atmosphere or to a low-pressure reservoir for sub-ambient pressure measurements. (b) Typical raw data showing the stepwise increase of the differential pressure and the resulting mass flow. By plotting steady-state flow against the differential pressure, plots were obtained from which permeability can be derived.

To study the gas permeability and predominant transport mechanism, the titania aerogels were sealed and analyzed in a custom-made permeability setup, as shown in Figure 3a. Permeability data were collected by applying multiple pressure gradients along the sample axis and recording the resulting steady-state volumetric flow (Figure 3b).

Steady-state methods require high-quality samples and proper sealing to provide accurate results. Cracks along the flow direction or gaps between the sample and sealant offer a path of least resistance and thus greatly affect the results. This is particularly problematic for materials with small pore sizes and therefore low permeability. For this study, a variety of sealants were evaluated, including different silicone rubbers, epoxy-based and solvent-based adhesives, hot-melt adhesives, and sealing wax. Among these, medium-viscosity RTV-1 silicone showed the best results in terms of surface adhesion and material compatibility (Figure S2). Unlike most of the other sealants, almost no change in the permeability was observed during repeated measurement cycles. Moreover, samples with different cross sections and thus circumferences showed almost identical permeabilities, implying a high sealing efficiency.

### Mass Transport Mechanism

Knowledge about the permeability and primary transport mechanism enables the identification and tackling of mass transfer limitations in catalytic processes and is essential for optimizing catalyst geometry, reactor design, and process conditions. In addition, permeability data can also be used to predict the structural properties of porous materials, such as pore size and connectivity, provided that the primary transport mechanism is known. To investigate the contribution of advection and diffusion to the overall mass transport through titania aerogels, we performed permeability measurements with different gases and average pressures. All tests were conducted on a single aerogel sample with an apparent density of 0.133 g/cm^3^ that was prepared by using a particle loading of 100 mg/mL and an acetone concentration of 54 vol %.

**Figure 4:**
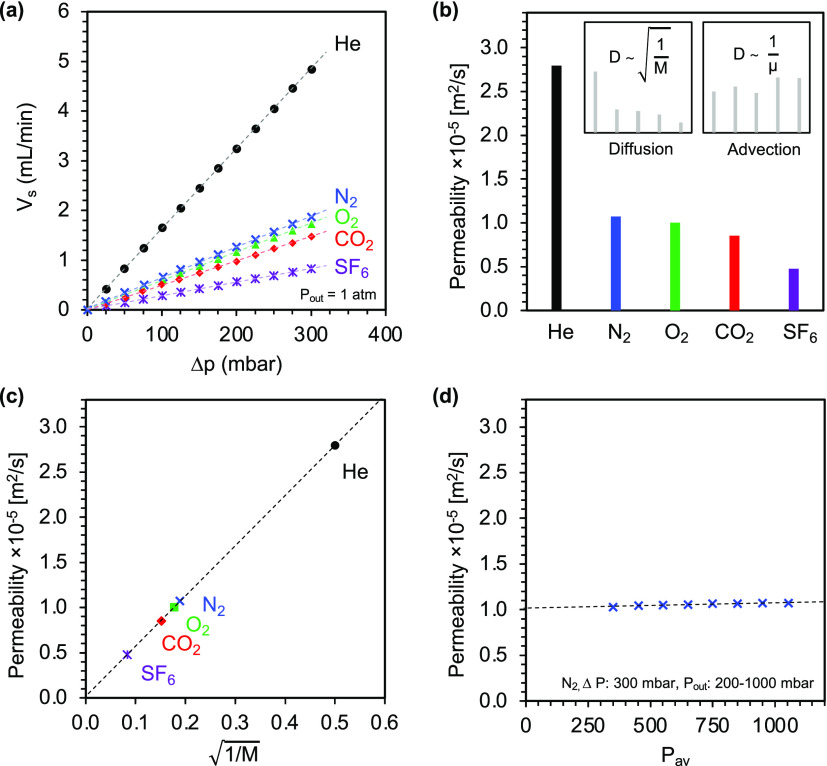
Permeability data of a single titania aerogel monolith for different gases and average pressures. (a) Linear relationship between applied differential pressure Δ*p* and the resulting steady-state mass flow rate *V*_s_ for different gases, with the slope being proportional to the permeability *D*. (b) Permeabilities for different gases compared to theoretical ratios for pure diffusive and advective flow. (c) Linear correlation between permeability and the inverse of the square root of the molar mass, indicating that mass transfer is purely diffusive. (d) Nitrogen permeability data obtained by applying a constant differential pressure of 300 mbar at varying in- and outlet pressures and thus average pressures. The permeability is independent of the average pressure, which is characteristic of mass transfer via Knudsen diffusion.

Figure 4a shows the steady-state flows of several gases at different differential pressures. In all cases, the mass flow increases linearly with the applied differential pressure. Permeabilities between 0.47 and 2.80 × 10^–5^ m^2^/s were found, which is in good agreement with reported permeability values for sol–gel-derived silica aerogels and carbon aerogels with similar porosities.^36,39,41,42^ Large deviations in permeability could be observed between helium and sulfur hexafluoride. This finding suggests that mass transport proceeds by diffusion rather than advection as these gases have very different molar masses but very similar dynamic viscosities. Figure 4b shows the permeabilities of different gases together with expected permeability ratios for pure diffusive and advective flow. The strong correlation between the permeability of different gases and their square root of molar mass (Figure 4c) is in perfect agreement with diffusion flow theory, implying that the mass transport is purely diffusive with no advective component. A similar correlation was also observed in studies on silica and carbon aerogels, where mass transport was attributed to Knudsen diffusion.^37,39,43^

Complementary experiments with constant differential pressure but varying input and output pressures revealed that the permeability is largely independent of the average pressure in the given pressure range (Figure 4d). This finding suggests that mass transport takes place primarily by Knudsen diffusion rather than ordinary diffusion, as the mean free path is not determined by molecule–molecule interactions but is restricted by collisions with the pore walls and therefore independent of the pressure. Moreover, contributions from advective mass transfer can be excluded, as advection would result in a strong correlation of permeability with average pressure (see eqs 2 and 3).

The independence of permeability on the average pressure is also reflected in the linear relationship of mass flow and pressure gradient observed in Figure 4a. Such linearity was observed across all samples analyzed in this study. However, most of the permeability data were collected at differential pressures between 0 and 300 mbar due to the limited mechanical stability of the lower loading aerogel samples. Some studies on sol–gel-derived silica and carbon aerogels reported a change in flow regime with increasing average pressures.^41,42^ To evaluate whether the linear relationship associated with Knudsen diffusion also holds at higher pressures, the differential pressure was stepwise increased to a maximum value of 1200 mbar at which point the sample failed. Only marginal deviations from linearity were observed between 300 and 1200 mbar, with permeabilities slightly lower than expected from interpolation between 0 and 300 mbar (<5%) (data not shown). A decrease in permeability might be attributed to a transition from Knudsen diffusion to ordinary diffusion because the mean free path length decreases at higher pressure, which increases the probability of molecule–molecule interactions. However, we believe that the observed deviation is more likely due to the compression of the aerogel under higher pressures, which reduces the average pore size and thus permeability.

### Effect of Gelation Conditions on Gas Permeability

**Figure 5:**
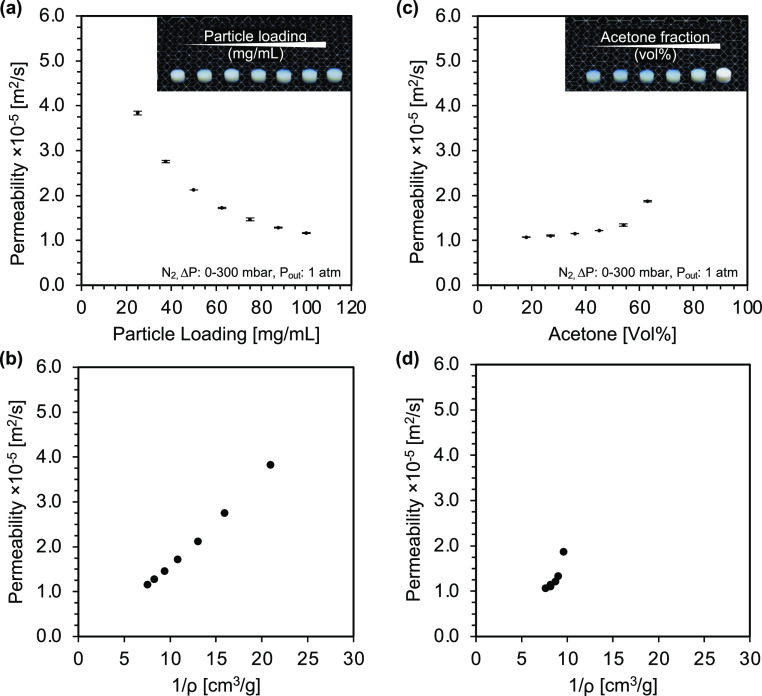
Effect of gelation parameters on the permeability of titania aerogels. Left: Permeability data of aerogels prepared with varying particle loadings (25–100 mg/mL) and constant acetone fraction (54 vol %). Right: Permeability data of titania aerogels were prepared by using a constant particle loading (75 mg/mL) and varying acetone fractions (18–63 vol %). For aerogels prepared with different particle loadings, the permeability is approximately inversely proportional to their apparent density, whereas for samples prepared with different acetone fractions, a nonlinear relationship can be found.

Figure 5 shows the permeability data of two series of titania aerogels prepared with different particle loadings (12–100 mg/mL) and acetone concentrations (18–63 vol %) for nitrogen. The samples were measured in triplicate, with several permeability curves recorded for each sample. Except for the sample with the lowest particle loading (12 mg/mL), which showed insufficient mechanical stability for proper sealing and analysis, the samples showed excellent repeatability over multiple permeability runs with only minor variations between similar samples (relative error <3%), indicating both high sealing efficiency and structural integrity.

The variation of particle loading during the gelation process has a strong influence on the permeability, as depicted in Figure 5a. This is expected because a lower particle concentration leads to higher porosity and thus higher permeability. When mass transfer is dominated by Knudsen diffusion, the permeability scales in a linear fashion with pore size. In an idealized network structure, the cross-sectional pore size and thus the permeability would double if half of the particles were removed. This is indeed the case, as permeability is approximately inversely proportional to particle loading, decreasing from 3.84 to 1.16 × 10^–5^ m^2^/s for particle loadings between 25 and 100 mg/mL. This relationship becomes even more apparent when the permeability is plotted against the apparent aerogel density (Figure 5c). An almost linear scaling was also reported for silica aerogels with similar densities as for this study,^39^ whereas for sintered silica aerogels and carbon aerogels, a power law dependence was found.^39,42^

In contrast, the destabilization strength affects the permeability much less, as shown in Figure 5b. Despite the very different optical properties, the permeability only varies between 1.07 and 1.87 × 10^–5^ m^2^/s for samples prepared with acetone fractions of 18–63 vol %. This is in line with expectations, as all samples were prepared with a constant particle loading and show only slight differences in density and porosity, which result from different shrinkage during the drying process. Although the permeability is relatively constant over a wide range, it sharply increases at higher acetone concentrations. This observation cannot be related to the variation in density and thus porosity, as seen in Figure 5d, but rather points to other structural changes. A possible origin of structural differences has already been discussed in the analysis of the optical properties. There we referred to the theory of cluster aggregation, according to which strong destabilization leads to more open cluster structures, which would also explain the increase in the permeability at higher acetone concentrations. To test this hypothesis, gas sorption measurements were performed, which will be discussed in the following.

### Gas Sorption Analysis

**Figure 6:**
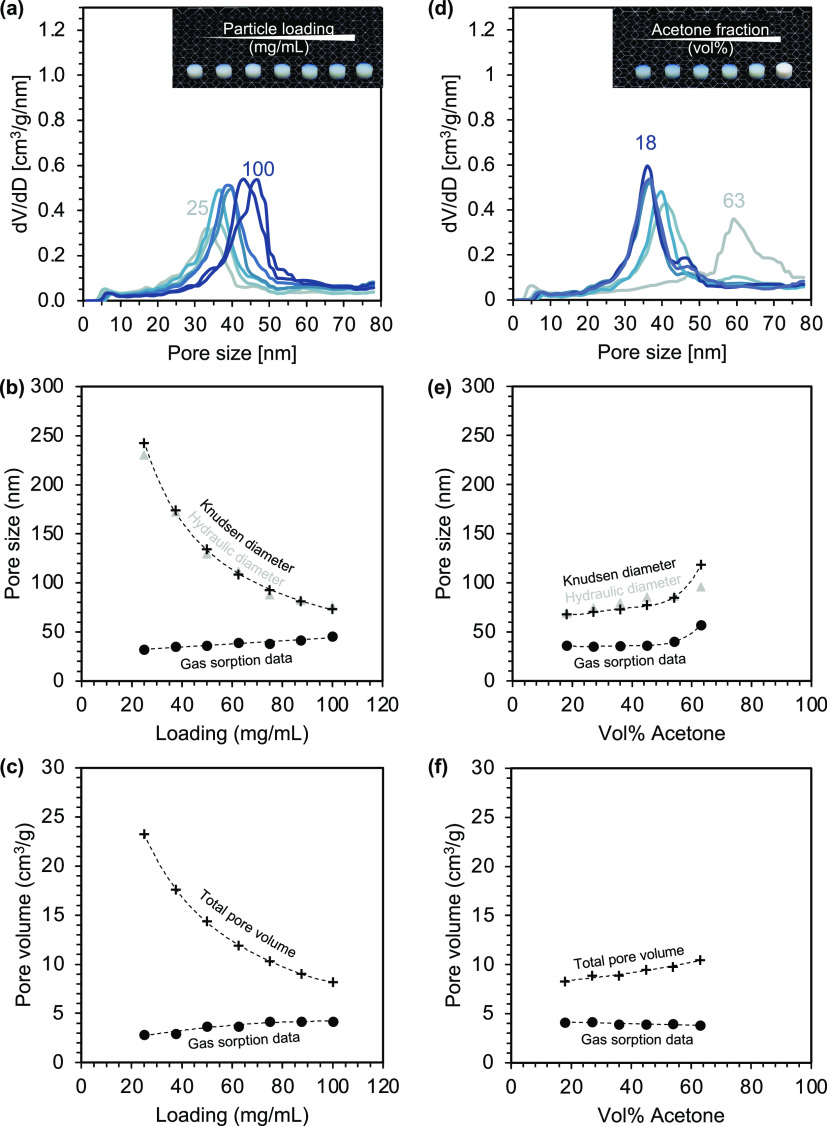
Nitrogen gas sorption data for aerogels prepared by varying particle loading (left) and acetone fraction (right) during the gelation process. (a, d) Pore size distribution determined by DFT analysis of gas sorption data. (b, e) Average pore size from gas sorption analysis compared to average pore diameter calculated from permeability data (Knudsen diameter) along with the average pore size estimated from the sample density, skeletal density, and specific surface area (hydraulic diameter, gray). (c, f) Pore volume determined by gas sorption analysis compared to total pore volume estimated from sample density and skeletal density.

To study the effect of the gelation parameters on the aerogel microstructure, nitrogen sorption measurements were performed on titania aerogels prepared with different particle loadings (25–100 mg/mL) and varying acetone fractions (18–63 vol %). All samples exhibit a type IV isotherm with a H3-hysteresis loop (see Figure S5, Supporting Information), which is attributed to a mesoporous structure with a nonuniform pore shape or pore size distribution. No substantial microporosity (pore size <2 nm) could be detected in the sorption data; however, the absence of a plateau at higher relative pressures indicates incomplete pore filling due to the presence of larger macropores. All samples exhibited similar specific surface areas around 450 m^2^/g (Table S2, Supporting Information), which agrees well with previous studies and is close to the theoretical limit for nanoparticles of 3–3.5 nm, indicating that the network is highly branched in all cases. The pore size distribution (Figure 6a,d) reveals a broad range of pore sizes extending from the mesoporous regime (2–50 nm) to the macroporous regime (≥50 nm). Most of the detected porosity arises from pores with sizes between 30 and 60 nm. Figure 6b,e shows the mean pore size from gas sorption analysis in comparison to the average pore size calculated from the permeability data (eqs 3 and 7) and the average pore size estimated from the sample density and specific surface area (eq 6).

For samples prepared by varying particle loadings, the mean pore sizes are in the range of 30–45 nm and thus significantly smaller than expected from permeability measurements (Figure 6b). We attribute this discrepancy partly to the presence of larger macropores (≥80 nm), that are not accounted for in gas sorption analysis. Evidence for such macropores was already found in the optical scattering experiments and was also revealed by microscopy images as discussed later, especially for networks formed at low particle concentrations. In the optical analysis, we hypothesized that the network formation likely proceeds via the formation of finely branched secondary clusters that further interconnect into a hierarchical network over time. A high particle fraction yields a high number of clusters and thus a comparably dense cluster packing, while a low particle content results in larger voids between the loosely packed clusters. Although gas sorption analysis cannot reveal such macroporosity, the measured mesoporosity fits well into this picture and can be attributed to the porosity within the secondary clusters. Their mean pore size is almost constant at different loadings, which is consistent with the theory of cluster aggregation, according to which similar destabilization strength leads to a similar cluster structure.

For samples prepared with different acetone fractions, the mean pore size follows the estimated value from the permeability data (Figure 6e). The pore size increases at higher acetone fractions which agrees well with the theory of cluster aggregation, predicting larger and more open porous clusters at higher destabilization strengths. The similarity between the two curves indicates that the gas permeability is governed by the structure of the secondary clusters. This finding can be explained by the high particle fraction used for this series (75 mg/mL) leading to a more densely packed network structure with rather low macroporosity. Nevertheless, the curves are significantly shifted, with the pore size from the permeability data being about twice as high as obtained by gas sorption analysis. The reasons for this shift can be manifold. For instance, both pore sizes are estimated assuming straight cylindrical pores with uniform cross sections. This assumption certainly does not reflect the intricate network structure of aerogels. Nonetheless, studies of gas flow through aerogels typically show good fits between experimental data and this simplified model.

To evaluate the accuracy of both methods, we compared the average pore size from the permeability and gas sorption data to the hydraulic diameter, an alternative measure of the pore size for channels with irregularly shaped cross sections, which is calculated by the sample density, skeletal density, and specific surface area. As shown in Figure 6b,e, the hydraulic diameter agrees well with the values derived from permeability studies, indicating that the gas sorption analysis significantly underestimates the pore size. Such underestimation of pore size is well known for aerogels, as capillary forces arising during pore filling and removal cause the finely branched network to contract.^57^ Studies on silica aerogels showed that such contraction can yield pore sizes that are 1.5–2 times smaller than those determined by other experimental techniques.^58^ A similar deviation can be observed for samples with higher particle loadings. However, samples with lower particle loadings show deviations of up to a factor of 10. Such a severe contraction would presumably cause the filigree microstructure to collapse, which we did not observe. Therefore, these large discrepancies are rather due to the fact that gas sorption analysis captures only micro-, meso-, and small macropores, thus missing the fraction of larger macropores that are also present in these samples. Accordingly, the resulting average pore sizes are smaller than the real ones, which are better mapped by the other two methods. To estimate the degree of macroporosity, the specific pore volume detected by gas sorption analysis was compared with the total pore volume calculated from the aerogel density (Figure 6c,f). Both series show high deviations between the determined and expected values. Accordingly, gas sorption analysis seems to account for only approximately 10% of the total pore volume for the lowest loading samples and about 50% for the highest loading samples. Studies on low-density silica aerogels showed comparable results, with only one-third of the total pore volume being detected by gas sorption analysis.^41^ However, we assume that the actual macroporosity is much smaller since gas sorption analysis tends to underestimate the pore volume similar to the pore size. This is due to the aforementioned volume contraction during gas sorption analysis, but also partly because of the inverse structure of aerogels, in which the adsorbate interface can adopt a zero curvature, suppressing pore condensation even in pores that are well within the mesoporous regime.^59,60^

From the gas sorption studies, we conclude that two levels of porosity exist within the aerogel microstructure. On the one hand, all samples show a well-defined pore size distribution with mean pore sizes of 35–60 nm, which we assign to mesoporosity within the secondary clusters. On the other hand, a comparison of detected and expected pore volumes indicates the presence of larger macropores, which we attribute to different packing densities of the secondary clusters. To verify this hypothesis, we examined aerogel samples prepared with different particle loadings and acetone fractions by scanning electron microscopy.

### Scanning Electron Microscopy

**Figure 7:**
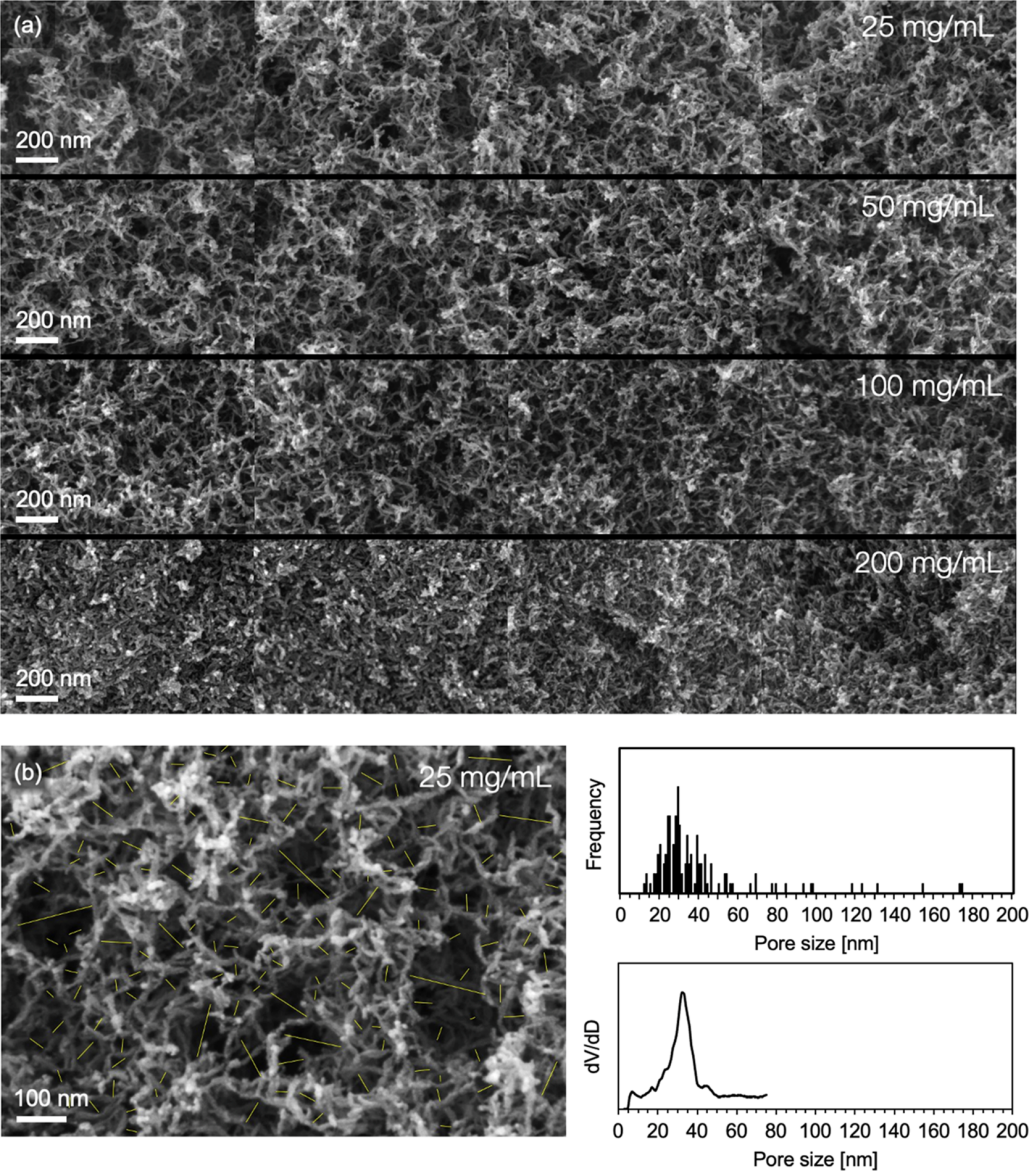
(a) Scanning electron micrographs of titania aerogels prepared with varying particle loadings at four different sample locations. For comparison, a sample with a particle loading of 200 mg/mL was prepared in addition to the samples of the series. (b) Magnified image of a 25 mg/mL sample showing the pearl-necklace-like structure. The overlay shows pores randomly selected for image analysis. (c) Pore size distribution from image analysis (top) in comparison to the pore size distribution obtained by nitrogen gas sorption analysis (bottom).

Figure 7a shows scanning electron microscopy (SEM) images of titania aerogels prepared with different particle loadings. The images confirm the presence of larger macropores with sizes up to 200–300 nm for the lowest particle loading. The large pores are surrounded by a finely branched pearl-necklace-like structure made up of individual titania nanoparticles (Figure 7b). The macropores decrease in size upon going to higher particle loadings and vanish for loadings above 100 mg/mL.

The pore size distribution obtained from single-image analysis is in good agreement with gas sorption data, as illustrated in Figure 7b,c, although the degree of macroporosity calculated from gas sorption data seems to be significantly overestimated for all samples, as discussed earlier. At particle loading below 50 mg/mL, the size of the macropores approaches the wavelength of visible light, which is in line with the enhanced scattering and the transition from Rayleigh to Mie scattering seen in the optical studies. Furthermore, the macropore sizes are similar to the pore size estimated from the permeability data. This finding suggests that mass transfer preferentially occurs through the larger pores, which is consistent with the theory of Knudsen diffusion.

**Figure 8:**
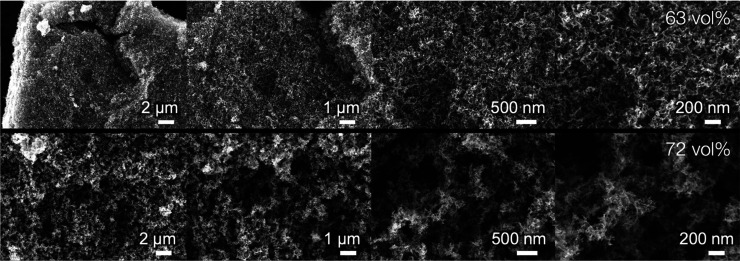
Scanning electron micrographs of titania aerogels prepared with different acetone fractions. Top: Microstructure of an opaque aerogel prepared with an acetone fraction of 63 vol %, showing a homogeneous texture without significant macroporosity. Bottom: Microstructure of a sample prepared with 72 vol % acetone, revealing a loose packing of relatively compact clusters of similar size.

For samples prepared with varying acetone fractions but constant particle loading, no obvious differences in the microstructure could be found in SEM images between 18 and 63 vol % acetone, although slight structural differences were evident in permeability and gas sorption data. A distinct change in microstructure was only observed for samples in which the acetone threshold of 63 vol % was exceeded. Figure 8 shows SEM images of the opaque sample prepared with 63 vol % acetone compared to a sample prepared with 72 vol % acetone exhibiting a chalklike appearance and poor mechanical properties. While 63 vol % acetone yields a finely branched homogeneous network, 72 vol % acetone results in a very loosely packed structure made up of rather compact aggregates. We believe that such a structure results from a collapse of finely branched clusters when the attraction between the branches of a cluster becomes too strong, which in turn implies that network formation indeed takes place via the formation of secondary clusters.

The high opacity of the 72 vol %-sample stems from macropores with sizes well above the wavelength of visible light. However, no such macroporosity could be found in SEM images for samples obtained with 18–63 vol % acetone. Moreover, all of these samples scatter in a Rayleigh-type fashion (see the Optical Characterization section and Figure S4). Therefore, we conclude that the change in opacity between 18 and 63 vol % acetone arises from more subtle changes, such as the increase in mesopore size found in gas sorption measurements. This seems plausible as Rayleigh scattering scales with the sixth power of the feature size so that even slight changes in mesopore size can cause strong changes in optical properties.

Overall, the observations from scanning electron microscopy are in good agreement with the results from optical studies, permeability measurement, and gas sorption analysis and confirm our hypothesis that the assembly of aerogels from presynthesized building blocks proceeds via the formation of secondary clusters that further interconnect to form a volume-filling gel. The destabilization strength primarily affects the mesoporous structure within the secondary clusters and thus the transparency, while the particle loading determines the packing density of the clusters and the void structure, which governs the mass transport rate in particular for lower particle loadings.

## Summary & Outlook

The preparation of aerogels by gelation of colloidal nanoparticle dispersions is an extremely versatile process, as it not only allows maximum flexibility in the selection of the particulate building blocks but also facilitates control over the microstructure during gelation. Compared to the well-established tuning of building blocks, little research has been devoted to controlling the microstructure during their gelation to regulate mass and light transport through centimeter-sized bodies.

Thanks to an optimized synthesis route giving access to high-quality titania aerogel monoliths with a wide range of porosities and transparency, we were able to study the gas permeability and light transmission in more detail and relate the macroscopic properties of the aerogels to their microstructure. Permeability measurements revealed that the mass transfer through these networks proceeds primarily via Knudsen diffusion, with the diffusion rate being mainly dependent on the porosity and thus particle loading used during gelation. In contrast, the optical properties can be controlled by adjusting the destabilization strength during gelation, which induces more subtle changes in the mesoporous structure that strongly impact the light transmittance but only slightly affect the permeability.

The ability to independently control mass and light transport offers great potential to tailor the properties of nanoparticle-based titania aerogels to specific needs, whereas the knowledge gained about the permeability and primary transport mechanism allows a systematic selection of photocatalyst geometries and reactor designs to minimize mass transport limitations and further improve the performance of titania aerogel photocatalysts.

## Supporting Information Available

Additional experimental details and numerical values for sample preparation, density determination, permeability measurements, and nitrogen gas sorption analysis (PDF)

## References

[ref1] Carroll M. K.; Anderson A. M. Aerogels as Platforms for Chemical Sensors. In Aerogels Handbook, Aegerter M. A.; Leventis N.; Koebel M. M., Eds.; Springer: Dordrecht, 2011; pp 637–650.

[ref2] Amonette J. E.; Matyas J. Functionalized Silica Aerogels for Gas-Phase Purification, Sensing, and Catalysis: A Review. Microporous Mesoporous Mater. 2017, 250, 100–119. 10.1016/j.micromeso.2017.04.055.

[ref3] Wan W. C.; Zhang R. Y.; Ma M. Z.; Zhou Y. Monolithic Aerogel Photocatalysts: A Review. J. Mater. Chem. A 2018, 6 (3), 754–775. 10.1039/C7TA09227J.

[ref4] Maleki H.; Hüsing N. Current Status, Opportunities and Challenges in Catalytic and Photocatalytic Applications of Aerogels: Environmental Protection Aspects. Appl. Catal., B 2018, 221, 530–555. 10.1016/j.apcatb.2017.08.012.

[ref5] Cai B.; Eychmüller A. Promoting Electrocatalysis upon Aerogels. Adv. Mater. 2019, 31 (31), e1804881 10.1002/adma.201804881. 30536681

[ref6] Pierre A. C.; Pajonk G. M. Chemistry of Aerogels and Their Applications. Chem. Rev. 2002, 102 (11), 4243–4266. 10.1021/cr0101306. 12428989

[ref7] Hüsing N.; Schubert U. Aerogels—Airy Materials: Chemistry, Structure, and Properties. Angew. Chem., Int. Ed. 2007, 37 (1–2), 22–45. 10.1002/(SICI)1521-3773(19980202)37:1/2<22::AID-ANIE22>3.0.CO;2-I 29710971

[ref8] Matter F.; Luna A. L.; Niederberger M. From Colloidal Dispersions to Aerogels: How to Master Nanoparticle Gelation. Nano Today 2020, 30, 100827 10.1016/j.nantod.2019.100827.

[ref9] Ziegler C.; Wolf A.; Liu W.; Herrmann A.-K.; Gaponik N.; Eychmüller A. Modern Inorganic Aerogels. Angew. Chem., Int. Ed. 2017, 56 (43), 13200–13221. 10.1002/anie.201611552. 28160389

[ref10] Bigall N. C.; Herrmann A. K.; Vogel M.; Rose M.; Simon P.; Carrillo-Cabrera W.; Dorfs D.; Kaskel S.; Gaponik N.; Eychmüller A. Hydrogels and Aerogels from Noble Metal Nanoparticles. Angew. Chem., Int. Ed. 2009, 48 (51), 9731–9734. 10.1002/anie.200902543. 19918827

[ref11] Herrmann A. K.; Formanek P.; Borchardt L.; Klose M.; Giebeler L.; Eckert J.; Kaskel S.; Gaponik N.; Eychmüller A. Multimetallic Aerogels by Template-Free Self-Assembly of Au, Ag, Pt, and Pd Nanoparticles. Chem. Mater. 2014, 26 (2), 1074–1083. 10.1021/cm4033258.

[ref12] Du R.; Fan X. L.; Jin X. Y.; Hubner R.; Hu Y.; Eychmüller A. Emerging Noble Metal Aerogels: State of the Art and a Look Forward. Matter 2019, 1 (1), 39–56. 10.1016/j.matt.2019.05.006.

[ref13] Georgi M.; Klemmed B.; Benad A.; Eychmüller A. A versatile ethanolic approach to metal aerogels (Pt, Pd, Au, Ag, Cu and Co). Mater. Chem. Front. 2019, 3 (8), 1586–1592. 10.1039/C9QM00193J.

[ref14] Cheng W.; Rechberger F.; Niederberger M. Three-Dimensional Assembly of Yttrium Oxide Nanosheets into Luminescent Aerogel Monoliths with Outstanding Adsorption Properties. ACS Nano 2016, 10 (2), 2467–2475. 10.1021/acsnano.5b07301. 26756944

[ref15] Rechberger F.; Ilari G.; Niederberger M. Assembly of Antimony-Doped Tin Oxide Nanocrystals into Conducting Macroscopic Aerogel Monoliths. Chem. Commun. 2014, 50 (86), 13138–13141. 10.1039/C4CC05648E. 25229075

[ref16] Rechberger F.; Ilari G.; Willa C.; Tervoort E.; Niederberger M. Processing of Cr Doped SrTiO_3_ Nanoparticles into High Surface Area Aerogels and Thin Films. Mater. Chem. Front. 2017, 1 (8), 1662–1667. 10.1039/C7QM00155J.

[ref17] Rechberger F.; Heiligtag F. J.; Süess M. J.; Niederberger M. Assembly of BaTiO_3_ Nanocrystals into Macroscopic Aerogel Monoliths with High Surface Area. Angew. Chem., Int. Ed. 2014, 53 (26), 6823–6826. 10.1002/anie.201402164. 24853124

[ref18] Cheng W.; Rechberger F.; Niederberger M. From 1D to 3D - Macroscopic Nanowire Aerogel Monoliths. Nanoscale 2016, 8 (29), 14074–14077. 10.1039/C6NR04429H. 27389477

[ref19] Rechberger F.; Tervoort E.; Niederberger M. Nonaqueous Sol-gel Synthesis of InTaO_4_ Nanoparticles and Their Assembly into Macroscopic Aerogels. J. Am. Ceram. Soc. 2017, 100 (10), 4483–4490. 10.1111/jace.15018.

[ref20] Heiligtag F. J.; Rossell M. D.; Suess M. J.; Niederberger M. Template-Free Co-Assembly of Preformed Au and TiO_2_ Nano- particles into Multicomponent 3D Aerogels. J. Mater. Chem. 2011, 21 (42), 16893–16899. 10.1039/c1jm11740h.

[ref21] Kashanchi G. N.; King S. C.; Ju S. E.; Dashti A.; Martinez R.; Lin Y. K.; Wall V.; McNeil P. E.; Marszewski M.; Pilon L.; Tolbert S. H. Using Small Angle X-ray Scattering to Examine the Aggregation Mechanism in Silica Nanoparticle-Based Ambigels for Improved Optical Clarity. J. Chem. Phys. 2023, 158 (3), 034702 10.1063/5.0130811. 36681626

[ref22] Deshmukh R.; Tervoort E.; Kach J.; Rechberger F.; Niederberger M. Assembly of Ultrasmall Cu_3_N Nanoparticles into Three-Dimensional Porous Monolithic Aerogels. Dalton Trans. 2016, 45 (29), 11616–11619. 10.1039/C6DT01451H. 27169877

[ref23] Hitihami-Mudiyanselage A.; Senevirathne K.; Brock S. L. Assembly of Phosphide Nanocrystals into Porous Networks: Formation of InP Gels and Aerogels. ACS Nano 2013, 7 (2), 1163–1170. 10.1021/nn305959q. 23346878

[ref24] Hitihami-Mudiyanselage A.; Senevirathne K.; Brock S. L. Bottom-Up Assembly of Ni_2_P Nanoparticles into Three-Dimensional Architectures: An Alternative Mechanism for Phosphide Gelation. Chem. Mater. 2014, 26 (21), 6251–6256. 10.1021/cm5030958.

[ref25] Mohanan J. L.; Brock S. L. A New Addition to the Aerogel Community: Unsupported Cds Aerogels with Tunable Optical Properties. J. Non-Cryst. Solids 2004, 350 (0), 1–8. 10.1016/j.jnoncrysol.2004.05.020.

[ref26] Mohanan J. L.; Arachchige I. U.; Brock S. L. Porous Semiconductor Chalcogenide Aerogels. Science 2005, 307 (5708), 397–400. 10.1126/science.1104226. 15662006

[ref27] Arachchige I. U.; Brock S. L. Sol–Gel Methods for the Assembly of Metal Chalcogenide Quantum Dots. Acc. Chem. Res. 2007, 40 (9), 801–809. 10.1021/ar600028s. 17441681

[ref28] Odziomek M.; Chaput F.; Lerouge F.; Dujardin C.; Sitarz M.; Karpati S.; Parola S. From Nanoparticle Assembly to Monolithic Aerogels of YAG, Rare Earth Fluorides, and Composites. Chem. Mater. 2018, 30 (15), 5460–5467. 10.1021/acs.chemmater.8b02443.

[ref29] Heiligtag F. J.; Airaghi Leccardi M. J.; Erdem D.; Süess M. J.; Niederberger M. Anisotropically Structured Magnetic Aerogel Monoliths. Nanoscale 2014, 6 (21), 13213–13221. 10.1039/C4NR04694C. 25255203

[ref30] Rechberger F.; Niederberger M. Translucent Nanoparticle-Based Aerogel Monoliths as 3-Dimensional Photocatalysts for the Selective Photoreduction of CO_2_ to Methanol in a Continuous Flow Reactor. Mater. Horiz. 2017, 4 (6), 1115–1121. 10.1039/C7MH00423K.

[ref31] Kwon J.; Choi K.; Schreck M.; Liu T.; Tervoort E.; Niederberger M. Gas-Phase Nitrogen Doping of Monolithic TiO_2_ Nanoparticle-Based Aerogels for Efficient Visible Light-Driven Photocatalytic H_2_ Production. ACS Appl. Mater. Interfaces 2021, 13 (45), 53691–53701. 10.1021/acsami.1c12579. 34730952

[ref32] Kwon J.; Choi K.; Tervoort E.; Niederberger M. One-Pot Microwave Synthesis of Pd Modified Titanium Dioxide Nanocrystals for 3D Aerogel Monoliths with Efficient Visible-Light Photocatalytic Activity in a Heated Gas Flow Reactor. J. Mater. Chem. A 2022, 10 (35), 18383–18395. 10.1039/D2TA04024G.

[ref33] Luna A. L.; Matter F.; Schreck M.; Wohlwend J.; Tervoort E.; Colbeau-Justin C.; Niederberger M. Monolithic Metal-Containing TiO_2_ Aerogels Assembled from Crystalline Pre-Formed Nanoparticles as Efficient Photocatalysts for H_2_ Generation. Appl. Catal., B 2020, 267, 118660 10.1016/j.apcatb.2020.118660.

[ref34] Schreck M.; Kleger N.; Matter F.; Kwon J.; Tervoort E.; Masania K.; Studart A. R.; Niederberger M. 3D Printed Scaffolds for Monolithic Aerogel Photocatalysts with Complex Geometries. Small 2021, 17 (50), e2104089 10.1002/smll.202104089. 34661959

[ref35] Matter F.; Niederberger M. The Importance of the Macroscopic Geometry in Gas-Phase Photocatalysis. Adv. Sci. 2022, 9 (13), 2105363 10.1002/advs.202105363. PMC9069382 35243811

[ref36] Stumpf C.; von Gässler K.; Reichenauer G.; Fricke J. Dynamic Gas Flow Measurements on Aerogels. J. Non-Cryst. Solids 1992, 145, 180–184. 10.1016/S0022-3093(05)80452-1.

[ref37] Beurroies I.; Bourret D.; Sempéré R.; Duffours L.; Phalippou J. Gas Permeability of Partially Densified Aerogels. J. Non-Cryst. Solids 1995, 186, 328–333. 10.1016/0022-3093(95)00084-4.

[ref38] Hasmy A.; Beurroies I.; Bourret D.; Jullien R. Gas Transport in Porous Media: Simulations and Experiments on Partially Densified Aerogels. Europhys. Lett. 1995, 29 (7), 567–572. 10.1209/0295-5075/29/7/010.

[ref39] Reichenauer G.; Stumpf C.; Fricke J. Characterization of SiO_2_, RF and Carbon Aerogels by Dynamic Gas Expansion. J. Non-Cryst. Solids 1995, 186, 334–341. 10.1016/0022-3093(95)00057-7.

[ref40] Satoh S.; Matsuyama I.; Susa K. Diffusion of Gases in Porous Silica Gel. J. Non-Cryst. Solids 1995, 190 (3), 206–211. 10.1016/0022-3093(95)00275-8.

[ref41] Hosticka B.; Norris P. M.; Brenizer J. S.; Daitch C. E. Gas Flow through Aerogels. J. Non-Cryst. Solids 1998, 225, 293–297. 10.1016/S0022-3093(98)00130-6.

[ref42] Kong F. M.; LeMay J. D.; Hulsey S. S.; Alviso C. T.; Pekala R. W. Gas Permeability of Carbon Aerogels. J. Mater. Res. 1993, 8 (12), 3100–3105. 10.1557/JMR.1993.3100.

[ref43] Reichenauer G.; Fricke J. Gas Transport in Sol-Gel Derived Porous Carbon Aerogels. MRS Proc. 1996, 464 (1), 345–350. 10.1557/PROC-464-345.

[ref44] Petričević R.; Glora M.; Fricke J. Planar Fibre Reinforced Carbon Aerogels for Application in PEM Fuel Cells. Carbon 2001, 39 (6), 857–867. 10.1016/S0008-6223(00)00190-1.

[ref45] Dullien F. A. L. Single-Phase Transport Phenomena in Porous Media. In Porous media: Fluid Transport and Pore Structure, Dullien F. A. L., Ed.; Academic Press: 1979; pp 157–234.

[ref46] O’Hanlon J. F. A User’s Guide to Vacuum Technology, 3rd ed.; John Wiley & Sons, 2003.

[ref47] Polleux J.; Pinna N.; Antonietti M.; Niederberger M. Ligand-Directed Assembly of Preformed Titania Nanocrystals into Highly Anisotropic Nanostructures. Adv. Mater. 2004, 16 (5), 436–439. 10.1002/adma.200306251.

[ref48] Polleux J.; Pinna N.; Antonietti M.; Hess C.; Wild U.; Schlögl R.; Niederberger M. Ligand Functionality as a Versatile Tool to Control the Assembly Behavior of Preformed Titania Nanocrystals. Chem.—Eur. J. 2005, 11 (12), 3541–3551. 10.1002/chem.200401050. 15736277

[ref49] Kotsokechagia T.; Cellesi F.; Thomas A.; Niederberger M.; Tirelli N. Preparation of Ligand-Free TiO_2_ (Anatase) Nanoparticles through a Nonaqueous Process and Their Surface Functionalization. Langmuir 2008, 24 (13), 6988–6997. 10.1021/la800470e. 18522445

[ref50] Akerlof G. Dielectric Constants of Some Organic Solvent-water Mixtures at Various Temperatures. J. Am. Chem. Soc. 1932, 54 (11), 4125–4139. 10.1021/ja01350a001.

[ref51] Korala L.; Brock S. L. Aggregation Kinetics of Metal Chalcogenide Nanocrystals: Generation of Transparent CdSe (ZnS) Core (Shell) Gels. J. Phys. Chem. C 2012, 116 (32), 17110–17117. 10.1021/jp305378u. PMC3439211 22984632

[ref52] Weitz D. A.; Huang J. S.; Lin M. Y.; Sung J. Limits of the Fractal Dimension for Irreversible Kinetic Aggregation of Gold Colloids. Phys. Rev. Lett. 1985, 54 (13), 1416–1419. 10.1103/PhysRevLett.54.1416. 10031026

[ref53] Lin M. Y.; Lindsay H. M.; Weitz D. A.; Ball R. C.; Klein R.; Meakin P. Universality in Colloid Aggregation. Nature 1989, 339 (6223), 360–362. 10.1038/339360a0.

[ref54] Jungblut S.; Joswig J. O.; Eychmüller A. Diffusion- and reaction-limited cluster aggregation revisited. Phys. Chem. Chem. Phys. 2019, 21 (10), 5723–5729. 10.1039/C9CP00549H. 30801102 PMC6484677

[ref55] Jungblut S.; Joswig J. O.; Eychmüller A. Diffusion-Limited Cluster Aggregation: Impact of Rotational Diffusion. J. Phys. Chem. C 2019, 123 (1), 950–954. 10.1021/acs.jpcc.8b10805. PMC6484677 30801102

[ref56] Mandal C.; Donthula S.; Soni R.; Bertino M.; Sotiriou-Leventis C.; Leventis N. Light Scattering and Haze in TMOS-co-APTES Silica Aerogels. J. Sol-Gel Sci. Technol. 2019, 90 (1), 127–139. 10.1007/s10971-018-4801-0.

[ref57] Scherer G. W.; Smith D. M.; Stein D. Deformation of Aerogels during Characterization. J. Non-Cryst. Solids 1995, 186, 309–315. 10.1016/0022-3093(95)00058-5.

[ref58] Reichenauer G.; Scherer G. W. Extracting the pore size distribution of compliant materials from nitrogen adsorption. Colloids Surf., A 2001, 187–188, 41–50. 10.1016/S0927-7757(01)00619-7.

[ref59] Scherer G. W. Adsorption in Sparse Networks: I. Cylinder Model. J. Colloid Interface Sci. 1998, 202 (2), 399–410. 10.1006/jcis.1998.5458.

[ref60] Scherer G. W.; Calas S.; Sempere R. Adsorption in Sparse Networks: II. Silica Aerogels. J. Colloid Interface Sci. 1998, 202 (2), 411–416. 10.1006/jcis.1998.5459.

