# Supplementary material for: Optimization of Mass and Light Transport in Nanoparticle-Based Titania Aerogels
Source: Chem Mater. 2023 Sep 20;35(19):7995–8008. doi: 10.1021/acs.chemmater.3c01218 (PMC10568969; doi:10.1021/acs.chemmater.3c01218)
Supplement: Supplementary file 1 — cm3c01218_si_001.pdf [file cm3c01218_si_001.pdf]

Optimization of Mass and Light Transport in Nanoparticle-based Titania Aerogels

*Fabian Matter, Markus Niederberger**

Laboratory for Multifunctional Materials, Department of Materials, ETH Zurich, Vladimir-Prelog-Weg 5, 8093 Zurich, Switzerland

SUPPORTING INFORMATION

Sample preparation

Table S1. Solvent ratios and gelation conditions used for the preparation of titania gels and aerogels with varying particle loadings and acetone fractions.

ID	Loading [mg/mL]	Acetone [vol%]	Dispersion [mL]	H_2O [mL]	Acetone [mL]	Temperature [°C]	Time [min]
L1	12.5	55	0.25	1.75	2.4	30	60
L2	25	55	0.5	1.5	2.4	30	60
L3	37.5	55	0.75	1.25	2.4	30	45
L4	50	55	1	1	2.4	30	45
L5	62.5	55	1.25	0.75	2.4	30	30
L6	75	55	1.5	0.5	2.4	30	30
L7	87.5	55	1.75	0.25	2.4	30	15
L8	100	55	2	0	2.4	30	15

ID	Loading [mg/mL]	Acetone [vol%]	Dispersion [mL]	H_2O [mL]	Acetone [mL]	Temperature [°C]	Time [min]
T1	75	18	1.5	2.1	0.8	50	60
T2	75	27	1.5	1.7	1.2	50	60
T3	75	36	1.5	1.3	1.6	40	60
T4	75	45	1.5	0.9	2	40	60
T5	75	54	1.5	0.5	2.4	30	30
T6	75	63	1.5	0.1	2.8	30	15

Density determination

The density of the specimen was calculated from its apparent mass and volume. The curvature of the meniscus was recorded by photographs, fitted to a circular segment (**Figure S1**), and included in the volume calculation as follows:

$$V_{monolith} = V_{cylinder} - V_{meniscus} = \pi \cdot \left(\frac{D}{2}\right)^2 \cdot L - \frac{\pi}{6} l \left(\frac{3}{4} D^2 + l^2\right)$$

with D and L being the diameter and height of the monolith and l the height of the spherical segment. The curvature of the opaque 63 vol%-sample was estimated by linear extrapolation of the height of the spherical segment.

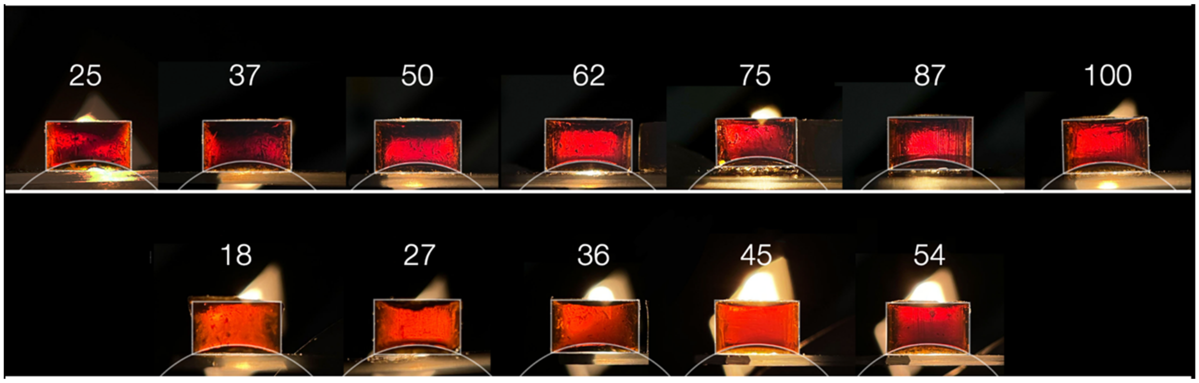

Figure S1. Backlight images of aerogel samples prepared with different particle loadings (25-100 mg/ml) (top) and different acetone fractions (18 – 54 vol%) (bottom) with the curvature of the meniscus fitted to a circular segment.

Permeability measurements

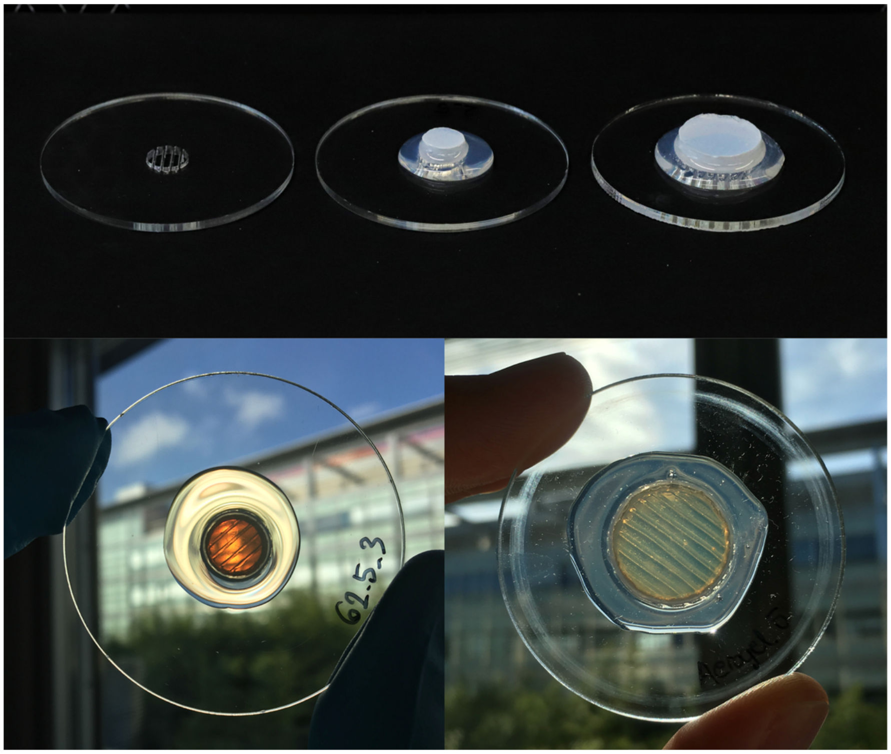

Figure S2. Perforated PMMA disc holder together with sealed titania aerogel samples of different diameters in side view and top view. Smaller aerogels were used for permeability measurements, the larger sample served as a reference for evaluating the sealing efficiency.

The meniscus formed during molding leads to variations in sample thicknesses along the gas flow direction, causing gas flow resistance to be higher at the outer edges than in the center. To account for this effect, a correction factor was used for the sample length. The factor was determined by comparing the theoretical permeability of a flat cylinder with that of an aerogel monolith of equal dimensions (Equations S1 – S4). For approximation, the monolithic body was divided into hollow tubes of variable radius and constant wall thickness, as shown in **Figure S2**, with a height profile of the photographically determined spherical segment. The permeability of each flow channel was calculated individually. The theoretical permeability was calculated by summing the permeability

of all flow channels. For opaque samples, the shape of the meniscus was estimated by extrapolating the curvature radius.

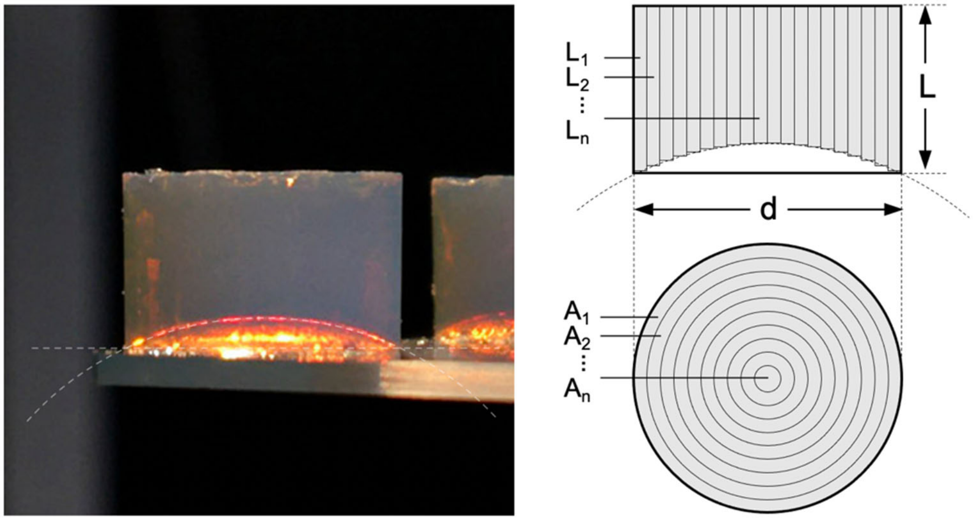

Figure S3. Left: Photographic determination of meniscus shape by fitting a circular segment to the curvature. Right: Approximation based on individual tubular flow channels with area A_n and height L_n used to estimate the relative permeability of aerogel monoliths.

$$\frac{pV}{A} = \mathrm{D} \cdot \frac{\Delta p}{L} \tag{S1}$$

$$\mathrm{D}_{\mathrm{cylinder}} = \frac{pV}{\Delta p} \cdot \frac{L}{A} \tag{S2}$$

$$\mathrm{D}_{\mathrm{monolith}} = \frac{\mathrm{pV}}{\Delta \mathrm{p}} \cdot \sum_{\mathrm{i}=1}^{\mathrm{n}} \frac{\mathrm{L_i}}{\mathrm{A_i}} = \frac{\mathrm{pV}}{\Delta \mathrm{p}} \cdot \frac{\mathrm{L}'}{\mathrm{A}} \tag{S3}$$

$$L' = L \cdot x \leftrightarrow x = \frac{L'}{L} = \frac{D_{monolith}}{D_{cylinder}} \approx 0.9 \tag{S4}$$

Optical analysis

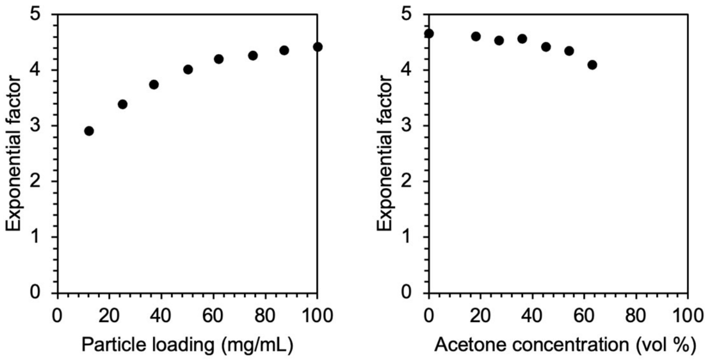

Figure S4. Exponential factor extracted from fitting the light attenuation data to a power law decay.

Gas sorption analysis

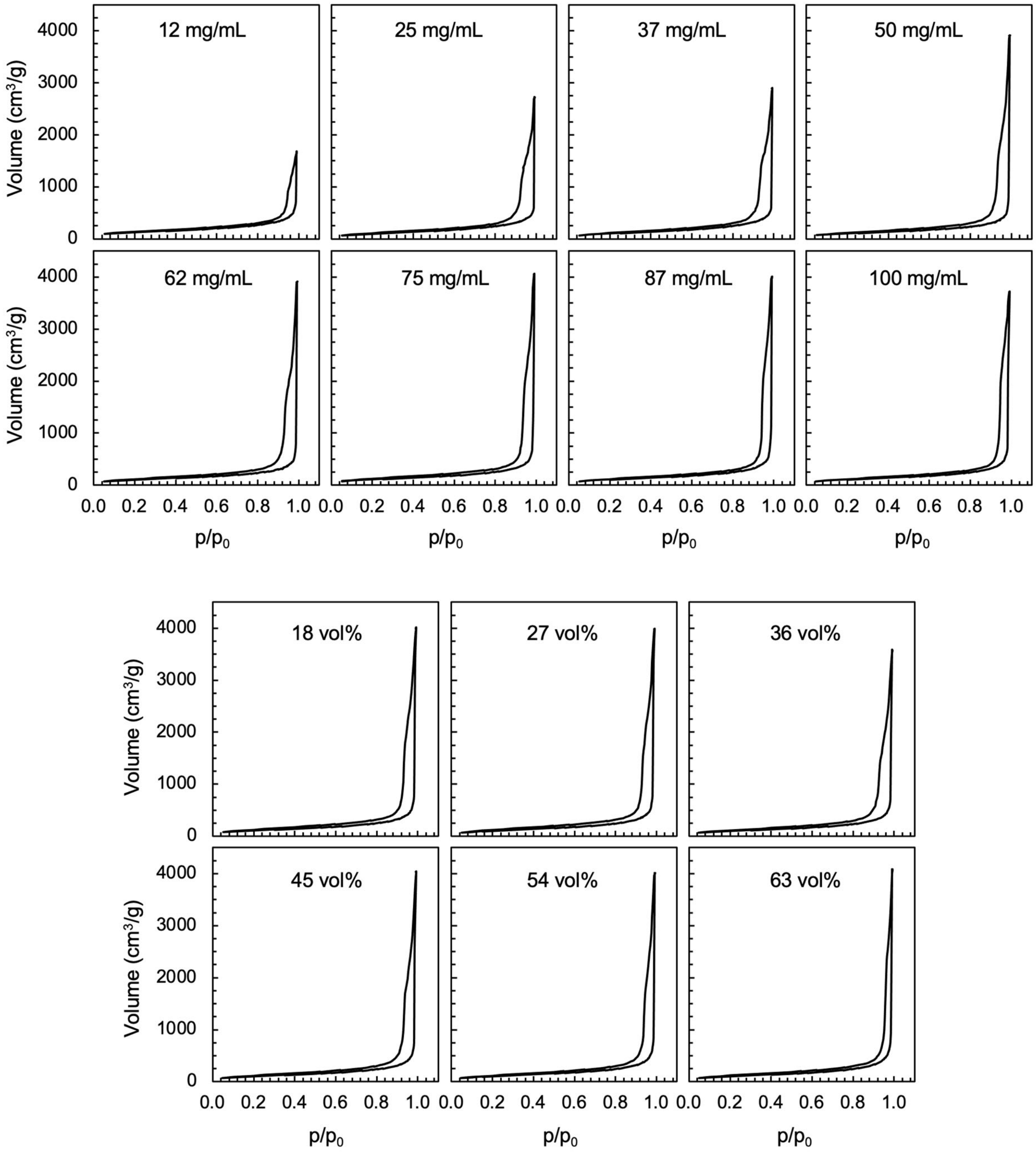

Figure S5. Gas sorption isotherms for titania aerogels prepared with varying particle loadings (top) and varying acetone fractions (bottom).

Table S2. Numerical values with corresponding standard deviations from density determination, permeability measurements, and nitrogen gas sorption analysis of titania aerogels prepared by different loadings and acetone fractions.

ID	Linear Shrinkage [%]	Density [mg/cm^3]	Porosity [%]	Permeability $\times 10^{-5}$ [m^2/s]	Surface area [m^2/g]	Pore vol. (DFT) [cm^3/g]	Pore vol. (calc.) [cm^3/g]	Pore size (DFT) [nm]	Pore size (Hydraulic) [nm]	Pore size (Permeability) [nm]
L2	17.9 ± 0.4	47.7 ± 1.5	98.77 ± 0.04	3.837 ± 0.146	404	2.83	23.28 ± 0.74	32.3	230.6 ± 7.3	242.4 ± 9.2
L3	14.2 ± 0.2	62.8 ± 1.8	98.38 ± 0.05	2.756 ± 0.090	409	2.96	17.62 ± 0.50	35.2	172.2 ± 4.9	174.1 ± 5.7
L4	12.4 ± 0.3	76.7 ± 1.7	98.03 ± 0.04	2.123 ± 0.043	444	3.69	14.38 ± 0.33	36.1	129.7 ± 2.9	134.1 ± 2.7
L5	11.3 ± 0.3	92.4 ± 2.3	97.62 ± 0.06	1.721 ± 0.041	425	3.70	11.90 ± 0.30	39.0	112.0 ± 2.8	108.8 ± 2.6
L6	10.2 ± 0.3	106.4 ± 2.2	97.26 ± 0.06	1.469 ± 0.060	467	4.20	10.30 ± 0.21	38.0	88.3 ± 1.8	92.8 ± 3.8
L7	9.4 ± 0.3	120.9 ± 2.2	96.89 ± 0.06	1.284 ± 0.030	437	4.20	9.04 ± 0.16	41.8	82.7 ± 1.5	81.1 ± 1.9
L8	8.6 ± 0.5	133.1 ± 2.4	96.58 ± 0.06	1.164 ± 0.027	430	4.16	8.19 ± 0.15	45.6	76.2 ± 1.4	73.5 ± 1.7

ID	Linear Shrinkage [%]	Density [mg/cm^3]	Porosity [%]	Permeability $\times 10^{-5}$ [m^2/s]	Surface area [m^2/g]	Pore vol. (DFT) [cm^3/g]	Pore vol. (calc.) [cm^3/g]	Pore size (DFT) [nm]	Pore size (Hydraulic) [nm]	Pore size (Permeability) [nm]
T1	16.1 ± 0.4	131.9 ± 3.9	96.61 ± 0.10	1.072 ± 0.017	477	4.14	8.26 ± 0.24	36.1	69.3 ± 2.0	67.8 ± 1.1
T2	15.3 ± 0.5	123.1 ± 2.0	96.84 ± 0.05	1.108 ± 0.041	476	4.16	8.87 ± 0.14	35.2	74.5 ± 1.2	70.0 ± 2.6
T3	14.2 ± 0.1	123.2 ± 1.1	96.83 ± 0.03	1.153 ± 0.005	446	3.93	8.86 ± 0.08	35.6	79.4 ± 0.7	72.9 ± 0.3
T4	13.3 ± 0.1	115.7 ± 2.4	97.03 ± 0.06	1.219 ± 0.006	442	3.93	9.46 ± 0.20	36.1	85.5 ± 1.8	77.0 ± 0.4
T5	12.2 ± 0.2	111.9 ± 2.2	97.12 ± 0.06	1.342 ± 0.046	453	3.94	9.79 ± 0.20	39.9	86.5 ± 1.7	84.8 ± 2.9
T6	10.5 ± 0.2	104.7 ± 1.4	97.31 ± 0.04	1.873 ± 0.043	437	3.80	10.48 ± 0.14	57.0	96.0 ± 1.3	118.3 ± 2.7
